# Dermoscopy as a Tool in Differentiating Cutaneous Squamous Cell Carcinoma From Its Variants

**DOI:** 10.5826/dpc.1102a50

**Published:** 2021-04-12

**Authors:** Dimitrios Sgouros, Melpomeni Theofili, Vasileia Damaskou, Sofia Theotokoglou, Konstantinos Theodoropoulos, Alexander Stratigos, Panagiotis Theofilis, Ioannis Panayiotides, Dimitrios Rigopoulos, Alexander Katoulis

**Affiliations:** 1 First Department of Dermatology & Venereology, Andreas Sygros Hospital, National and Kapodistrian University of Athens, School of Medicine, Athens, Greece; 2 Second Department of Dermatology & Venereology, Attikon General University Hospital, National and Kapodistrian University of Athens, School of Medicine, Athens, Greece; 3 Second Department of Pathology, Attikon General University Hospital, National and Kapodistrian University of Athens, School of Medicine, Athens, Greece; 4 Department of Internal Medicine, General Hospital of Nikaia Agios Panteleimon, Piraeus, Greece

**Keywords:** skin tumors, Bowen disease, keratoacanthoma, invasive squamous cell carcinoma, dermoscopy

## Abstract

**Background:**

Dermoscopic features of cutaneous squamous cell carcinoma (cSCC) have been widely studied, but their accuracy should be further investigated.

**Objectives:**

This study assessed the diagnostic accuracy of a set of predetermined dermoscopic structures for 3 variants of cSCC, namely Bowen disease, keratoacanthoma and invasive cSCC.

**Methods:**

Dermoscopic images of 56 histopathologically confirmed cSCC lesions (9 Bowen disease lesions, 7 keratoacanthomas, and 40 invasive cSCCs) were examined, and the diagnostic accuracy of dermoscopic structures was assessed. Discriminative ability of statistically significant positive predictors was determined using receiver operating characteristic (ROC) curves, and defined as an area under the ROC curve >0.700.

**Results:**

Dermoscopic structures with statistical significance and discriminative ability were: for Bowen disease, clustered glomerular vessels and erosions; for keratoacanthoma, a central keratin plug; and for invasive cSCC, a mixed color of the background. Clustered and glomerular vessels had, for Bowen disease, perfect diagnostic accuracy, with: sensitivity of 88.9% for both features; specificity of 97.9% and 93.6%, respectively; positive predictive value (PPV) of 88.9% and 72.7%, respectively; and negative predictive value (NPV) of 97.8% for both. Erosions had, for BD, high specificity (87.2%) and NPV (91.1%), but low sensitivity (55.6%) and PPV (45.5%). A central keratin plug had, for keratoacanthoma, high specificity (87.8%) and NPV (93.5%), but low sensitivity (57.1%) and PPV (40%). A mixed background color had, for invasive cSCC, high specificity (81.3%) and PPV (89.7%), but low sensitivity (65%) and NPV (48.2%).

**Conclusion:**

Dermoscopy accurately differentiates BD, through clustered glomerular vessels, from keratoacanthoma and invasive cSCC. Dermoscopic structures of keratoacanthoma and invasive cSCC overlap, and only histopathologic analysis differentiates them precisely.

## Introduction

Cutaneous squamous cell carcinoma (cSCC) is the second most prevalent skin cancer [1]. Common risk factors for the development of cSCC are cumulative sun exposure, ionizing radiation, immunosuppression, chronic skin inflammation and a family history of cSCC [2]. The 3 main clinical subtypes of cSCC are Bowen disease (BD), keratoacanthoma and invasive cSCC.

Dermoscopy is a noninvasive clinical tool that allows the identification of morphological characteristics of an examined lesion, not seen by the naked eye or with a magnifying lens. Use of dermoscopy facilitates early diagnosis and the good clinical management of skin lesions [3]. In addition, dermoscopy contributes to the treatment of skin tumors by enabling the preoperative determination of tumor surface boundaries, assessment of the effects of local therapies, and postoperative follow-up of patients [4].

Prespecified dermoscopic features of BD include pink or pigmented background, clustered glomerular vessels (coiled vessels, mimicking the glomerular apparatus of the kidney), dotted vessels (like small red dots), opaque yellow-white scales, and erosions [4–9]. Dermoscopic criteria for keratoacanthoma are a white background, central keratin plug (amorphous, yellow-white to light brown), white circles around follicular openings, and vascular loops that are hairpin-like or made of linear irregular vessels [6,10]. Dermoscopic features of invasive cSCC depend on the state of differentiation: Highly differentiated invasive cSCC has a background that is white, mixed (white to pink, red to white, pink to red) or pigmented, and demonstrates hairpin-like or linear irregular vessels, keratin clods (amorphous masses of keratin, yellow-white to light-brown), white circles around follicular openings, and ulcerations (red or red to brown structureless areas) [4,6,11,12]. Poorly differentiated invasive cSCC exhibits a red or mixed (red to white, pink to red) background, a polymorphous vascular pattern with more than one vessel type dominating (consisting of linear irregular, hairpin-like, glomerular and, rarely, dotted types), and ulcerations [4,6,11,12].

Considering the ability of invasive cSCC to metastasize if left untreated, the early differential diagnosis of cSCC variants is very important [1,13]. Despite the importance of dermoscopy in the initial clinical evaluation, data concerning the diagnostic accuracy of dermoscopic criteria of cSCC variants are limited.

## Methods

### Study Population

We enrolled 53 patients with 56 cSCCs (9 BD, 7 keratoacanthomas, 40 invasive cSCCs) from the Dermato-Oncology Unit of the Second Department of Dermatology-Venerology of Attikon University Hospital in Athens, Greece. Inclusion criteria were: (i) a histopathologic diagnosis of BD, keratoacanthoma or invasive cSCC (basosquamous or metatypical basal cell carcinoma were excluded from the study) following complete surgical excision of the tumor; and (ii) a high quality dermoscopic image. All individuals gave written informed consent after being informed about the purposes of the study. The study was approved by the Ethics Committee of Attikon University Hospital.

### Clinical and Anthropometric Measurements

Initially and without knowledge of the histopathologic diagnosis, we recorded the demographic characteristics and the history of the patients. Specifically, the patients were questioned about the presence of risk factors associated with the development of non-melanoma skin cancers, such as sun exposure habits, sunburns, sunscreen usage and a history of basal cell carcinoma or cSCC, as well as a full medical history. Next, a thorough dermatologic examination was performed by the dermatologist.

### Dermoscopic Examination

Dermoscopic images were obtained using contact dermoscopy with a DermLite II Pro hybrid dermatoscope (×10) coupled to a Nikon J1 camera. Image evaluation was performed by 2 independent examiners (D.S., A.K.) blinded to the histopathologic diagnosis and considering predetermined dermoscopic criteria of cSCCs. In brief, vascular structures were scored according to their morphology (dotted, glomerular, hairpin-like, linear irregular, polymorphous) and arrangement (diffuse, clustered, peripheral). Moreover, the examiners assessed the presence of keratinized structures (scales, keratin clods, central keratin plug, white circles around follicular openings), erosions, and ulcerations, and recorded the color of the lesion background (pink, red, white, pigmented, or mixed).

### Statistical Analysis

Continuous variables were checked for normality of distribution by visual inspection of P-P plots and are presented as mean ± SD. Categorical variables are displayed as percentages. One-way analysis of variance (ANOVA) was used for comparisons between continuous and categorical variables. Differences between categorical variables were tested by forming contingency tables and performing χ^2^ tests. Statistically significant results were considered when P < 0.05. Then, the discriminative ability (accuracy) of the statistically significant positive predictors was assessed through the analysis of ROC (receiver operating characteristics) curves. The accuracy of dermoscopic criteria was estimated by calculating the area under the ROC curve (AUROC score). In addition, sensitivity, specificity, and positive and negative predictive values (PPV and NPV) of significant dermoscopic criteria with AUROC score >0.700 were calculated according to standard formulas [14]. All statistical calculations were performed using SPSS software (version 25.0; SPSS Inc., Chicago, Illinois, USA).

## Results

### Patients’ Demographics

The study considered 56 cSCCs from 53 patients (Table 1). Specifically, there were 9 BD lesions, 7 keratoacanthomas, and 40 invasive cSCCs (in 38 patients); 1 patient had both BD + invasive cSCC. Invasive cSCC lesions tended to be found in older patients who were more likely to have had unintentional sun exposure (invasive cSCC, 27 of 40 lesions (67.5%); BD, 4/9 (44.4%); keratoacanthoma, 2/7 (28.6%), P = .10). They also were more likely found in patients who reported a lack of sunscreen use (invasive cSCC, 38/40 (95.0%); BD, 7/9 (77.8%); keratoacanthoma, 5/7 (71.4%), P = .08). No significant differences were noticed regarding the frequency of sunburns before the age of 18, intentional sun exposure, or a smoking habit.

### Clinical Features of cSCC

Concerning patients with BD and keratoacanthoma, the most common site of occurrence of the lesions was the upper limb; these lesions were often hyperkeratotic (Table 2). A nodular surface was a characteristic finding in all keratoacanthomas, while BD lesions were mainly flat-like plaque. Solar lentigo in sun-exposed parts of the body and actinic keratosis also tended to be present upon inspection of both lesion types (Table 2). The invasive cSCC lesions were mostly located on the head and neck. They were mainly nodular and ulcerated. Moreover, actinic keratosis and solar lentigo in sun-exposed body parts were frequently noted (Table 2).

### Dermoscopic Features of cSCC

**Figure 1:**
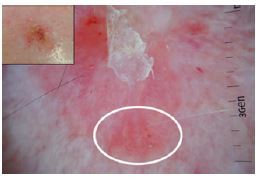
Clustered glomerular vessels (white circle) over an evenly colored pink background with slight scaling are the most striking findings in Bowen disease.

The dermoscopic features of cSCC are presented in Table 3. With regards to vascular structures, clustered glomerular vessels (found in 8 of 9 BD lesions (88.9%), 1 of 7 keratoacanthomas (14.3%), 3 of 40 invasive cSCC (7.5%), P < .001) were present in the vast majority of BD lesions (Figure 1). Linear irregular vessels were observed mostly in keratoacanthoma and invasive cSCC (BD, 1/9 (11.1%); keratoacanthoma, 4/7 (57.1%); invasive cSCC, 24/40 (60.0%), P = .03). Polymorphous vessels were detected in some keratoacanthomas and invasive cSCC (BD, 0/9; keratoacanthoma, 1/7 (14.3%); invasive cSCC, 10/40 (25%), P = .14). Dotted and hairpin-like vessels were uncommon in our sample. A peripheral vascular distribution was identified in keratoacanthomas and invasive cSCCs, but it was more frequent in keratoacanthomas (BD, 0/9; keratoacanthoma, 5/7 (71.4%); invasive cSCC, 23/40 (57.5%), P = .004). A diffuse vascular arrangement was more often seen in invasive cSCC without, however, reaching significance (BD, 1/9 (11.1%); keratoacanthoma, 1/7 (14.3%); invasive cSCC, 16/40 (40%), P = .14).

**Figure 2:**
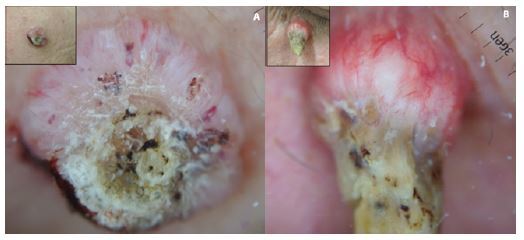
Well-differentiated (A) squamous cell carcinoma and (B) keratoacanthoma sharing common clinical and dermoscopic features. Pinkish white background along with linear irregular vessels and a central keratin plug are typical of both clinical entities.

Concerning features of keratinization, the presence of a central keratin plug was documented in the majority of keratoacanthomas and in a small proportion of invasive cSCC (BD, 0/9; keratoacanthoma, 5/7 (57.1%); invasive cSCC, 5/40 (12.5%), P = .004) (Figure 2, A and B). Scales, white circles around follicular openings, and keratin clods were found with similar frequencies in all the examined lesion types.

The presence of erosions was significantly associated with BD, while only a minor portion of invasive cSCC had this finding (BD, 5/9 (55.6%); keratoacanthoma, 2/7 (28.6%); invasive cSCC, 4/40 (10%), P = .007). On the contrary, ulcerations were mostly seen in invasive cSCCs (BD, 4/9 (44.4%); keratoacanthoma, 3/7 (42.9%); invasive cSCC, 32/40 (80%), P = .03).

Consequently, examination of the background color revealed a significant correlation of white with keratoacanthomas (BD, 1/9 (11.1%); keratoacanthoma, 2/7 (28.6%); invasive cSCC, 1/40 (2.5%), P = .04) and a predominance of a mixed color in invasive cSCCs (BD, 0/9; keratoacanthoma, 3/7 (42.9%); invasive cSCC, 26/40 (65%), P = .002). Importantly, those colors were rarely seen in BD lesions. Pink predominated in BD without, however, reaching statistical significance (BD, 5/9 (55.6%); keratoacanthoma, 1/7 (14.3%); invasive cSCC, 8/40 (20%), P = .07). Pigmented lesions were not identified among keratoacanthomas.

**Figure 3:**
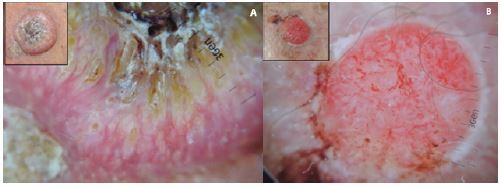
The dermoscopic progression of invasive squamous cell carcinoma. (A) Keratin clods surrounded by white circles, linear irregular vessels heading to the center of the tumor, and pinkish white background are typical characteristics of a highly differentiated squamous cell carcinoma. (B) Pronounced ulceration with diffusely arranged polymorphous vessels and prevalent red coloration are dermoscopic criteria for a poorly differentiated squamous cell carcinoma.

**Figure 4:**
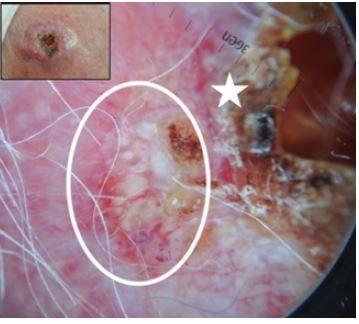
Intermediate differentiation of invasive squamous cell carcinoma. Admixed features of high differentiation such as white circles, keratin clods, and centrifugal linear vessels (white circle), and signs of low differentiation such as erosions and ulcerations (white star).

Table 4 presents the dermoscopic features of highly, intermediately, and poorly differentiated invasive cSCCs (Figures 3 and 4). White perifollicular openings were significantly associated with highly differentiated cSCC.

### Diagnostic Significance of Dermoscopic Structures

The AUROC scores of dermoscopic structures for the examined variants of cSCC are presented in Table 5. Regarding BD, clustered and glomerular vascular structures had excellent scores (>0.900) and hence excellent discriminative abilities, while erosions had a good score (>0.700) and discriminative ability. Pink background and scales had weak scores and therefore a poor discriminative ability for BD lesions. The discriminative capacity of the central keratin plug of keratoacanthomas was good, while those of a peripheral vascular arrangement and white background were poor. For invasive cSCC, a diffuse vascular arrangement and ulcerations had weak discriminative ability, while a mixed color of the background had a good discriminative potential.

For dermoscopic criteria with AUROC >0.700, the sensitivity, specificity, PPV and NPV were calculated (Table 6). Among dermoscopic findings of BD, glomerular vessels, clustered vessels and erosions had high specificity (93.6%, 97.9%, 87.2%, respectively) and NPV (97.8%, 97.9% 91.1%, respectively). The glomerular and clustered vessels had high sensitivity (88.9% for both), while erosions had low sensitivity (55.6%). PPV was high for clustered vessels (88.9%), moderate for glomerular vessels (72.7%), and poor for erosions (45.5%). A central keratin plug for keratoacanthoma had high specificity and NPV (87.8% and 93.5%, respectively), but its sensitivity was low (57.1%) and its PPV was weak (40%). Finally, a mixed color of the background for invasive cSCCs displayed high specificity (81.3%) and PPV (89.7%), but low sensitivity and NPV (65% and 48.2%, respectively).

## Discussion

This study evaluated the accuracy of various predetermined dermoscopic criteria for the early diagnostic assessment of cSCCs. Interesting results were obtained concerning the vascular patterns of the lesions. Clustered glomerular vessels were a potent predictor of BD and peripheral vessels were weakly correlated to keratoacanthoma, as were linear irregular vessels to invasive cSCCs.

Analytically, glomerular vessels were significantly associated with BD, as they were much more frequent in BD than in keratoacanthoma or invasive cSCC, in agreement with previous studies [3, 5–9, 15–19]. They had diagnostic accuracy for BD when the differential diagnosis was BD versus keratoacanthoma and invasive cSCC, with high sensitivity, specificity and NPV, in addition to moderate PPV. Moreover, a clustered vascular arrangement was the most valuable dermoscopic clue for BD, consistent with other reports [3,20]. It revealed high sensitivity, specificity, PPV and NPV for BD. It is notable that only one BD lesion had a diffuse, linear irregular vascular pattern instead of a clustered glomerular pattern, but the histopathologic diagnosis was BD progressing to invasive cSCC.

Regarding keratoacanthoma and invasive cSCC, the vascular pattern could not accurately differentiate them [12]. Invasive cSCCs, highly and poorly differentiated, commonly displayed linear irregular vessels, but with weak discriminative ability. Indeed they were detected at a similar frequency in keratoacanthoma (57.1% vs. 60% in invasive cSCC). Dotted, hairpin-like and polymorphous vessels were not significant features. Dotted and hairpin-like vessels were scarcely observed in keratoacanthoma and invasive cSCC, while polymorphous vessels were seen in some invasive cSCCs of intermediate and low differentiation, in accordance with previous research [12]. A peripheral vascular distribution was more prominent in keratoacanthoma than in invasive cSCC (71.4% and 57.5%, respectively) [12]. However, the latter had a poor diagnostic accuracy, as specificity for keratoacanthoma was low. On the other hand, a diffuse vascular distribution did not reach statistical significance.

Concerning keratinized structures, the presence of a central keratin plug should be interpreted carefully. It should be considered suggestive of keratoacanthoma since it was encountered approximately in half of these lesions, but it was completely absent from BD and was also observed in a small proportion of highly differentiated invasive cSCCs. It had high specificity for keratoacanthoma but low sensitivity. Therefore, a diagnosis of keratoacanthoma cannot be made with certainty upon its detection. This result is in line with previous dermoscopic studies [12,21]. Furthermore, scales, keratin clods, and white perifollicular circles failed to meet statistical significance. Scales and keratin clods were frequent in BD (77.8% and 66.7%, respectively), but they were also present in approximately half of keratoacanthomas and invasive cSCCs. White circles were more common in keratoacanthoma and invasive cSCC (85.7% and 70%, respectively), but were also found in approximately half of BD lesions. The later was an unexpected finding according to existing literature, and suggests that white circles are a strong indicator of keratoacanthoma and invasive cSCC. This finding is probably because white circles correspond to changes induced by orthokeratosis and parakeratosis or to an optical effect produced by the interaction of keratin-filled follicular openings with the polarized light of the dermoscope [12, 22]. Moreover, white circles were significantly associated with highly differentiated invasive cSCCs, in synchrony with earlier data [11].

Erosions and ulcerations were significant dermoscopic features, but with weak diagnostic accuracy. Erosions were more commonly observed in BD than in keratoacanthoma (approximately 2-fold higher) and in invasive cSCC (approximately 5-fold higher) [5–9]. They had, for BD, good discriminative ability, high specificity, and NPV, but low sensitivity and PPV. Ulcerations were found in the majority of invasive cSCCs compared to BD and keratoacanthomas (approximately 2-fold higher for both) [4, 6]. They had a poor discriminative ability for invasive cSCC. Furthermore, it is important to note that ulcerations observed with the unaided eye and dermoscopically in keratoacanthomas and invasive cSCCs were equally frequent.

Finally, analysis of the background color of the lesions revealed further significant features: white color for keratoacanthoma, and mixed color for invasive cSCC [20]. The white color displayed a weak discriminative potential for keratoacanthoma since it was quite frequent in keratoacanthomas and rare in BD and invasive cSCC (28.6% vs. 11.1% and 2.5% respectively). Moreover, a mixed background color exhibited a good discriminative capacity for invasive cSCC. It was present in 65% of invasive cSCCs and 42.9% of keratoacanthomas, but was completely absent in BD. This finding is possibly due to neo-vascularization of the malignant neoplasm in addition to the degree of differentiation, given that keratoacanthoma is considered by some dermatopathologists to be a highly differentiated invasive cSCC [23]. It has high specificity and PPV for invasive cSCC and low sensitivity and NPV. The pink background color observed in approximately half of BD exhibited a trend toward significance (P = .07). The pigmented background was absent from keratoacanthoma, in line with a previous study [12]. Therefore, the background of the lesion offers supplementary clinical evidence, but it could not aid the efficient dermoscopic differential diagnosis of cSCC variants.

The primary limitation of our study consists of the relatively small number of examined lesions, a fact that could impact the strength of our results. Second, the suggested accuracy of dermoscopic criteria cannot be generalized, but refers only to the differential diagnosis between the 3 variants of cSCC. Furthermore, adequately powered studies ought to be performed to confirm our findings and improve the knowledge surrounding the overall importance of dermoscopy.

## Conclusions

The presence of clustered glomerular vessels is a highly accurate dermoscopic criterion in differentiating, rapidly and efficiently, BD from keratoacanthoma and invasive cSCC, and may enhance the clinical diagnosis of BD in everyday practice. When contrasting keratoacanthoma from invasive cSCC, dermoscopic features overlap and should lead to consideration of biopsy and histopathologic analysis.

## Figures and Tables

**Table 1:** Demographic Characteristics for 56 Cutaneous Squamous Cell Carcinomas From 53 Patients

	Bowen Disease (*n* = 9)	Keratoacanthoma (*n* = 7)	Invasive cSCC (*n* = 40)	*P*
Age, mean ± SD, y	72.2 ± 10.2	73.3 ± 12.4	79.1 ± 9.7	.14
Male sex, n (%)	6 (66.7)	3 (42.9)	28 (70.0)	.38
Sunburn before age 18, n (%)	3 (33.3)	1 (14.3)	10 (25.0)	.68
Intentional sun exposure, n (%)	9 (100)	7 (100)	36 (90.0)	.42
Unintentional sun exposure, n (%)	4 (44.4)	2 (28.6)	27 (67.5)	.10
Lack *of sunscreen, n (%)*	7 (77.8)	5 (71.4)	38 (95.0)	.08
Smoking, n (%)	6 (66.7)	3 (42.9)	13 (32.5)	.16
Immunosuppression, n (%)	3 (33.3)	1 (14.3)	16 (40.0)	.42
cSCC history, n (%)	3 (33.3)	0 (0)	10 (25.0)	.26
BCC history, n (%)	0 (0)	1 (14.3)	4 (10.0)	.55

BCC = basal cell carcinoma; BD = Bowen disease; cSCC = cutaneous squamous cell carcinoma.

**Table 2:** Clinical Characteristics and Macroscopic Features of 56 Lesions

Characteristic^a^	BD (*n* = 9)	Keratoacanthoma (*n* = 7)	Invasive cSCC (*n* = 40)	*P*
Actinic keratosis	7 (77.8)	5 (71.4)	38 (95.0)	.03
SL (SE body parts)	7 (77.8)	5 (71.4)	36 (90.0)	.06
SL (NSE body parts)	0 (0)	2 (28.6)	12 (30.0)	.42
**Fitzpatrick phototype**				
II	4 (44.4)	1 (14.3)	13 (32.5)	.26
III	5 (55.6)	6 (85.7)	20 (50.0)	
IV	0 (0)	0 (0)	7 (17.5)	
**Lesion location**				
Head and neck	1 (11.1)	1 (14.3)	34 (85.0)	<.001
Trunk	3 (33.3)	1 (14.3)	3 (7.5)	
Upper limb	4 (44.4)	4 (57.1)	3 (7.5)	
Lower limb	1 (11.1)	1 (14.3)	0 (0)	
**Lesion morphology**				
Flat-like plaque	6 (75.0)	0 (0)	7 (17.5)	.001
Raised nodule	2 (25.0)	7 (100)	33 (82.5)	
No erosion-ulceration	3 (33.3)	4 (57.1)	5 (12.5)	.03
Erosion	2 (22.2)	0 (0)	3 (7.5)	
Ulceration	4 (44.4)	3 (42.9)	32 (80.0)	
Hyperkeratosis	6 (66.7)	5 (71.4)	22 (55.0)	.46

a Values are n (%).

BD = Bowen disease; cSCC = cutaneous squamous cell carcinoma; NSE = not sun-exposed; SE = sun-exposed; SL = solar lentigo.

**Table 3:** Dermoscopic Features of 56 cSCC by Histopathologic Diagnosis

Characteristic^a^	BD (n = 9)	Keratoacanthoma (n = 7)	Invasive cSCC (n = 40)	P
Vascular structures				
Dotted	0 (0)	0 (0)	1 (2.5)	.82
Hairpin-like	0 (0)	1 (14.3)	2 (5.0)	.45
Glomerular	8 (88.9)	1 (14.3)	3 (7.5)	<.001
Linear irregular	1 (11.1)	4 (57.1)	24 (60.0)	.03
Polymorphous	0 (0)	1 (14.3)	10 (25.0)	.22
**Vascular arrangement**				
Diffuse	1 (11.1)	1 (14.3)	16 (40.0)	.14
Clustered	8 (88.9)	1 (14.3)	0 (0)	<.001
Peripheral	0 (0)	5 (71.4)	23 (57.5)	.004
**Features of keratinization**				
Scales	7 (77.8)	4 (57.1)	23 (57.5)	.52
Keratin clods	6 (66.7)	3 (42.9)	23 (57.5)	.63
White circles	5 (55.6)	6 (85.7)	28 (70.0)	.43
Central keratin plug	0 (0)	4 (57.1)	5 (12.5)	.004
**Erosions and ulcerations**				
None	0 (0)	2 (28.6)	4 (10.0)	.18
Erosions	5 (55.6)	2 (28.6)	4 (10.0)	.007
Ulcerations	4 (44.4)	3 (42.9)	32 (80.0)	.03
**Background color**				
White	1 (11.1)	2 (28.6)	1 (2.5)	.04
Pink	5 (55.6)	1 (14.3)	8 (20)	.07
Red	2 (22.2)	1 (14.3)	4 (10)	.60
Pigmented	1 (11.1)	0 (0)	1 (2.5)	.39
Mixed	0 (0)	3 (42.9)	26 (65)	.002

a Values are n (%).

BD = Bowen disease; cSCC = cutaneous squamous cell carcinoma.

**Table 4:** Dermoscopic Features of 40 Invasive cSCC by Histopathologic Degree of Differentiation

Characteristic^a^	High (*n* = 21)	Intermediate (*n* = 12)	Low (*n* = 7)	*P*
**Vascular structures**				
Dotted	1 (4.8)	0 (0)	0 (0)	.63
Hairpin-like	1 (4.8)	1 (8.3)	0 (0)	.72
Glomerular	3 (14.3)	0 (0)	0 (0)	.23
Linear irregular	12 (57.1)	7 (58.3)	5 (71.4)	.79
Polymorphous	4 (19.0)	4 (33.3)	2 (28.6)	.64
**Vascular arrangement**				
Diffuse	9 (42.9)	7 (58.3)	0 (0)	.04
Clustered	0 (0)	0 (0)	0 (0)	-
Peripheral	11 (52.4)	5 (41.7)	7 (100)	.04
**Features of keratinization**				
Scales	15 (71.4)	6 (50.0)	2 (28.6)	.11
Keratin clods	13 (61.9)	7 (58.3)	3 (42.9)	.68
White circles	18 (85.7)	8 (66.7)	2 (28.6)	.02
Central keratin plug	4 (19.0)	1 (8.3)	0 (0)	.37
**Erosions and ulcerations**				
None	3 (14.3)	1 (8.3)	0 (0)	.54
Erosions	3 (14.3)	1 (8.3)	0 (0)	.54
Ulcerations	15 (71.4)	10 (83.3)	7 (100)	.25
**Background color**				
White	1 (4.8)	0 (0)	0 (0)	.63
Pink	4 (19.0)	3 (25.0)	1 (14.3)	.84
Red	2 (9.5)	2 (16.7)	0 (0)	.50
Pigmented	1 (4.8)	0 (0)	0 (0)	.63
Mixed	13 (61.9)	7 (58.3)	6 (85.7)	.44

a Values are n (%).

**Table 5:** AUROC Scores of Dermoscopic Structures

	BD (*n* = 9)	Keratoacanthoma (*n* = 7)	Invasive cSCC (*n* = 40)
**Vascular structures**			
Dotted	0.489	0.490	0.513
Hairpin-like	0.468	0.551	0.494
Glomerular	0.902	0.459	0.256
Linear irregular	0.258	0.531	0.644
Polymorphous	0.383	0.469	0.594
**Vascular arrangement**			
Diffuse	0.375	0.398	0.638
Clustered	0.934	0.490	0.219
Peripheral	0.202	0.631	0.622
**Features of keratinization**			
Scales	0.602	0.480	0.444
Keratin clods	0.557	0.418	0.506
White circles	0.416	0.592	0.506
Central keratin plug	0.404	0.735	0.438
**Erosions and ulcerations**			
None	0.436	0.602	0.488
Erosions	0.714	0.551	0.331
Ulcerations	0.350	0.347	0.681
**Background color**			
White	0.524	0.622	0.419
Pink	0.682	0.439	0.413
Red	0.558	0.510	0.456
Pigmented	0.545	0.480	0.481
Mixed	0.191	0.449	0.731

AUROC = area under the receiver operating characteristic curve; BD = Bowen disease; cSCC = cutaneous squamous cell carcinoma.

**Table 6:** Diagnostic Accuracy of Dermoscopic Criteria for cSCC

Dermoscopic Criterion	Sensitivity	Specificity	PPV	NPV
**BD**				
Glomerular vascular structures	88.9%	93.6%	72.7%	97.8%
Clustered vascular structures	88.9%	97.9%	88.9%	97.9%
Erosions	55.6%	87.2%	45.5%	91.1%
**Keratoacanthoma**				
Central keratin plug	57.1%	87.8%	40%	93.5%
**Invasive cSCC**				
Mixed background	65%	81.3%	89.7%	48.2%

BD = Bowen disease; cSCC = cutaneous squamous cell carcinoma; NPV = negative predictive value; PPV = positive predictive value.
